# Non-invasive prenatal diagnosis of spinal muscular atrophy by relative haplotype dosage

**DOI:** 10.1038/ejhg.2016.195

**Published:** 2017-01-25

**Authors:** Michael Parks, Samantha Court, Benjamin Bowns, Siobhan Cleary, Samuel Clokie, Julie Hewitt, Denise Williams, Trevor Cole, Fiona MacDonald, Mike Griffiths, Stephanie Allen

**Affiliations:** 1 West Midlands Regional Genetics Laboratory, Birmingham Women's NHS Foundation Trust, Birmingham, UK; 2 West Midlands Regional Genetics Service, Birmingham Women's NHS Foundation Trust, Birmingham, UK

## Abstract

Although technically possible, few clinical laboratories across the world have implemented non-invasive prenatal diagnosis (NIPD) for selected single-gene disorders, mostly owing to the elevated costs incurred. Having previously proven that NIPD for X-linked disorders can be feasibly implemented in clinical practice, we have now developed a test for the NIPD of an autosomal-recessive disorder, spinal muscular atrophy (SMA). Cell-free DNA was extracted from maternal blood and prepared for massively parallel sequencing on an Illumina MiSeq by targeted capture enrichment of single-nucleotide polymorphisms across a 6 Mb genomic window on chromosome 5 containing the SMN1 gene. Maternal, paternal and proband DNA samples were also tested for haplotyping purposes. Sequencing data was analysed by relative haplotype dosage (RHDO). Six pregnant SMA carriers and 10 healthy pregnant donors were recruited through the NIPSIGEN study. Inheritance of the maternally and paternally derived alleles of the affected SMN1 gene was determined in the foetus by RHDO analysis for autosomal-recessive disorders. DNA from the proband (for SMA carriers) or an invasively obtained foetal sample (for healthy pregnant donors) was used to identify the maternal and paternal reference haplotypes associated with the affected SMN1 gene. Results for all patients correlated with known outcomes and showed a testing specificity and sensitivity of 100%. On top of showing high accuracy and reliability throughout the stages of validation, our novel test for NIPD of SMA is also affordable and viable for implementation into clinical service.

## Introduction

The presence of cell-free foetal DNA (cffDNA) in maternal plasma during pregnancy was first described in 1997.^1^ Apoptosis of placental trophoblasts releases small fragments of cffDNA, which enter the maternal circulation.^2^ These fragments comprise approximately 10% of the total cell-free DNA (cfDNA) in maternal blood,^3, 4^ with the remainder being maternally derived. The discovery of cffDNA presented significant opportunities in terms of prenatal diagnostics. Methodologies based around the detection of paternally inherited sequences are well established, with clinical uses including foetal sexing^5, 6, 7^ and RhD blood group genotyping.^8, 9, 10^ Advances in massively parallel sequencing (MPS) technologies have led to the development of non-invasive prenatal testing (NIPT) for aneuploidy screening,^11, 12, 13, 14, 15^ which is now an established service in many countries worldwide.^16^ Methods for the non-invasive prenatal diagnosis (NIPD) of single-gene disorders (SGDs) have also been developed^17, 18^ but have been mostly limited to the detection of paternally inherited^19, 20, 21^ and *de novo* mutations.^22^ However, recent studies have proven that MPS-based approaches for NIPD of SGDs, including *β*-thalassemia,^23^ congenital adrenal hyperplasia (CAH)^24, 25^ and Duchenne and Becker muscular dystrophies (DMD/BMD),^26^ are technically possible but prohibitively expensive when considering clinical implementation.

Our group recently published the results of a study conducted at the Birmingham Women's NHS Foundation Trust (UK) as part of the NIPSIGEN project (Non-Invasive Prenatal diagnosis for Single Gene disorders) aimed at the development and clinical implementation of affordable NIPD tests for SGDs. Our method, which uses capture-based targeted enrichment followed by MPS and analysis by relative haplotype dosage (RHDO),^27^ enabled the highly accurate NIPD of DMD/BMD in at-risk pregnancies at an affordable cost for clinical implementation.^28^ We have now extended this method to encompass NIPD of spinal muscular atrophy (SMA), with similarly promising results.

SMA is an autosomal-recessive neurodegenerative disease with variable expression, caused by deletions, gene conversions or mutations in the SMN1 gene.^29, 30^ With an incidence of around 1 in 10 000 live births, it is the second most common autosomal-recessive disorder after cystic fibrosis in the northern European population.^31^ SMN1 maps to a complex region of chromosome 5q13.1, where an element of around 500 kb containing four genes (SMN, NAIP, SERF and GTFH2) is present in inverted duplicate copies. SMN1, located in the telomeric copy, is almost identical to the pseudogene SMN2, located in the centromeric copy, except for five nucleotide changes in exons 7 and 8.^30^ Causative mutations are divided into three categories: (1) homozygous deletion of SMN1 or gene conversion from SMN1 to SMN2 (approximately 95% of SMA cases); (2) compound heterozygosity involving deletion/gene conversion of one copy of SMN1 with a nonsense, frameshift or missense mutation in the remaining copy (approximately 5% of cases); and (3) homozygous or compound heterozygous mutation of SMN1 (very rare).^31^ Prenatal diagnosis of at-risk pregnancies currently involves the analysis of foetal DNA obtained by invasive procedures, such as chorionic villus sampling (CVS) or amniocentesis. SMN1 copy number can be determined by methods, including restriction fragment length polymorphism testing, multiplex ligation-dependent probe amplification (MLPA) or quantitative PCR.^31^ However, this type of analysis is limited in cases where two or more copies of SMN1 are present *in cis* on the same chromosome in combination with SMN1 deletion/gene conversion on the other chromosome, which accounts for around 4% of SMA carriers.^32^ Other intragenic mutations can be detected by sequencing or linkage analysis using informative markers flanking SMN1.^31^ Obtaining samples by these types of invasive techniques is the only diagnostic option currently available and is associated with a 0.5–1% risk of miscarriage.^33, 34^ An alternative non-invasive strategy would eliminate this risk and allow testing to be carried out at an earlier point in gestation. Our method for NIPD of SMA has shown promising results during validation testing, with a level of affordability conducive to clinical implementation.

## Subjects and methods

### Patients and samples

Patients were recruited into two separate groups through the NIPSIGEN study (‘NIPSIGEN: clinical translation of NIPD for SGDs' REC approval number: 13/NW/0580). Group 1 included pregnant women referred owing to risk of foetal aneuploidy who were offered invasive prenatal testing at West Midlands Regional Genetics Laboratory. Blood samples from women and their partners in this group were used as validation controls to assess the efficiency, accuracy and multiplexing capacity of our method. NIPD testing by RHDO can be performed on these patients by using the foetal genomic DNA obtained through invasive sampling to determine the reference haplotypes. Pregnant couples who are known carriers of SMA mutations were recruited nationwide (UK) to group 2. For these patients, we requested a sample of genomic DNA from a previous affected child to use as proband in determining the haplotypes linked with the paternal and maternal mutant alleles. The DNA samples needed for each patient included the cfDNA extracted from maternal plasma, the maternal and paternal genomic DNA extracted from leukocytes and the proband genomic DNA (from the invasively obtained sample for group 1 patients and from a previous affected child for group 2 patients). A maximum of 12 samples (ie, up to three patients) were processed simultaneously and pooled together prior to targeted capture enrichment and MPS. cfDNA was extracted from 4 ml of plasma and eluted in a final volume of 60 *μ*l. Maternal genomic DNA was extracted from the leukocytes contained in 1 ml of the blood cell portion, with paternal genomic DNA extracted in the same manner. Foetal genomic DNA was extracted from enzymatically digested CVS/amniotic fluid material and eluted into a final volume of 50 *μ*l. Proband DNA samples were extracted externally. Full details on sample processing and DNA extraction can be found in Supplementary Appendix SA.

### Targeted MPS

DNA libraries for MPS on the MiSeq sequencing platform (Illumina, Inc., San Diego, CA, USA) were prepared from 29 to 122 ng of input DNA. Capture enrichment was designed to target highly heterozygous single-nucleotide polymorphisms (SNPs) across the SMN1/SMN2 gene region (Chr 5: 67 000 530–72 999 964). Up to 12 samples (equivalent to up to three patients) were multiplexed per sequencing run using 2 × 80 cycles paired-end settings. More details can be found in Supplementary Appendix SB. Bioinformatics analysis included quality trimming of reads, alignment to genome build hg19, removal of duplicates and variant calling to obtain SNP counts (Supplementary Appendix SC).

### Data analysis by RHDO

RHDO analysis for autosomal-recessive disorders has been successfully used in proof-of-concept studies for NIPD of *β*-thalassemia^23^ and CAH.^24^ In this study, we have applied RHDO analysis for NIPD of SMA in a similar manner. Briefly, both maternal and paternal haplotypes linked with the mutant alleles were identified by DNA sequencing of highly heterozygous bi-allelic SNPs on both sides of the SMN1 gene (as previously described) in a previous affected child. The genotypes of the same SNPs were determined in maternal and paternal DNA samples to conduct haplotype phasing and identify the haplotypes linked with the normal alleles. SNP counts obtained from cfDNA sequencing were used to determine whether the foetus had inherited the mutated (M-ma) or normal (N-ma) maternal alleles and the mutated (M-pa) or normal (N-pa) paternal alleles. The paternally inherited haplotype was identified by using SNPs that were homozygous in the mother and heterozygous in the father. The maternally inherited haplotype was determined by RHDO analysis conducted on SNPs that were heterozygous in the mother and homozygous in the father. In this instance, SNPs had to be further subdivided into *α* (where the paternal SNP allele matched the maternal SNP allele on the affected haplotype) and *β* SNPs (where the paternal SNP allele matched the maternal SNP allele on the normal haplotype) and were analysed separately. The foetal fraction was determined by using SNPs that were homozygous in both parents but for different SNP alleles (Supplementary Appendix SD). Informative SNPs used to determine maternal and paternal inheritance were separately grouped into haplotype blocks of ≥25 SNPs to form maternal *α* and *β* blocks and paternal blocks. Each haplotype block represents a statistically independent result. Data quality filters and RHDO analysis parameters were adopted from previous publications^24, 28^ (Supplementary Appendix SDinformation).

### MLPA analysis

Routine invasive prenatal diagnosis of SMA patients was conducted using MLPA analysis to detect copy number of exons 7 and 8 in the SMN1 and SMN2 genes (SALSA MLPA P021 SMA kit, sold by MRC-Holland (Amsterdam, The Netherlands)). The genomic coordinates of the exons tested are the following: SMN1 exon 7: chr5.hg19:g.70 247 768–70 247 821; SMN1 exon 8: chr5.hg19:g.70 248 266–70 248 839; SMN2 exon 7: chr5.hg19:g.69 372 348–69 372 401; SMN2 exon 8: chr5.hg19:g.69 372 846–69 373 422.

## Results

### Probe selection criteria for efficient SNP enrichment

**Figure 1:**
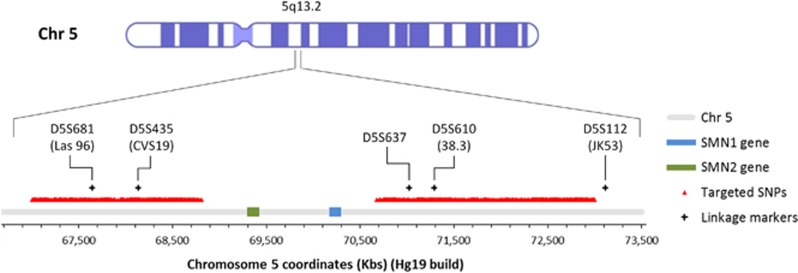
Diagram of the 5q13.2 locus on chromosome 5 containing the SMN1 and SMN2 genes represented by the light blue and green boxes, respectively. The red triangles indicate the chromosome position of SNPs with AvHet>0.4, which were targeted through capture-based DNA library enrichment for NIPD of SMA. The black crosses indicate the position of the markers routinely used in our laboratory for linkage analysis in SMA families.

Targeted enrichment by probe capture prior to MPS has been successfully used for copy number variation tests in combination with RHDO analysis in previous studies.^23, 24^ More recently, we were successful in further developing this approach by designing a highly efficient probe library for the targeted capture of >1000 SNPs across the dystrophin gene for NIPD testing of DMD/BMD.^28^ Using the same criteria, we selected 3039 SNPs from dbSNP(144) with a reported average heterozygosity of ≥40% across a 6 Mb region on chromosome 5 (Chr5: 67 000 530–72 999 964) containing the SMN1 and SMN2 genes (1000 genomes data). Probes were then specifically designed to uniquely target the selected SNPs. A highly repetitive region exists within the targeted 6 Mb (see Figure 1); therefore, SNPs within this range (Chr5: 68 813 676–70 680 481) were excluded from the probe design. The overall captured area was 276 Kb and SNPs were evenly distributed between the centromeric (1486 SNPs) and telomeric (1553 SNPs) regions flanking the SMN1 and SMN2 genes.

### Initial validation on group 1 patients: pregnancies A–J

Samples of healthy patients undergoing prenatal diagnosis for aneuploidy testing owing to increased risk (recruited locally to group 1) were initially used as controls to ascertain the efficiency, accuracy and multiplexing capacity of the test. Blood samples from the 10 patients tested were taken between 11 and 15 weeks of gestation. Details on outcomes and testing parameters are summarised in Table 1. On average, we observed a foetal fraction of 10.93% (ranging between 6.83 and 20.31%) and identified 632 informative SNPs per patient (interquartile range (IQR)=250) after quality filtering. The quantity of haplotype blocks identified for maternal inheritance ranged from 3 to 20, while haplotype blocks identified for paternal inheritance ranged from 4 to 12. Overall, 216/217 haplotype blocks were correctly classified, resulting in a testing accuracy of 99.53%. For these patients, the maternal and paternal reference haplotypes identified from the corresponding DNA sample obtained by CVS or amniocentesis were classified as HapA-ma and HapA-pa, respectively. The alternative haplotypes identified by phasing in maternal and paternal DNA samples were classified as HapB-ma and HapB-pa. Therefore, we expected to observe an outcome of HapA-ma/HapA-pa in all tested pregnancies of group 1 patients, which was indeed the case. The results reported in Table 1 demonstrate that the test was capable of delivering high accuracy and consistency for group 1 patients, with an overall sensitivity and specificity of 100% and a maximum multiplexing capacity of three patients per sequencing run (MiSeq sequencing platform (Illumina, Inc.). A single maternal haplotype block was incorrectly classified in family F but did not affect the final result. Incorrect classification of haplotype blocks in RHDO analysis has been previously reported at an estimated rate of <2%^27^ and can be caused by low foetal fraction and underlying biological factors. Therefore, a switch in inheritance pattern observed in a single haplotype block is disregarded and does not affect the final result. However, two or more consecutive haplotype blocks showing a switch in inheritance pattern indicate the presence of a recombination event,^24, 27, 28^ which needs to be taken into account in the final result.

### Results for group 2 patients: families K–O

**Figure 2:**
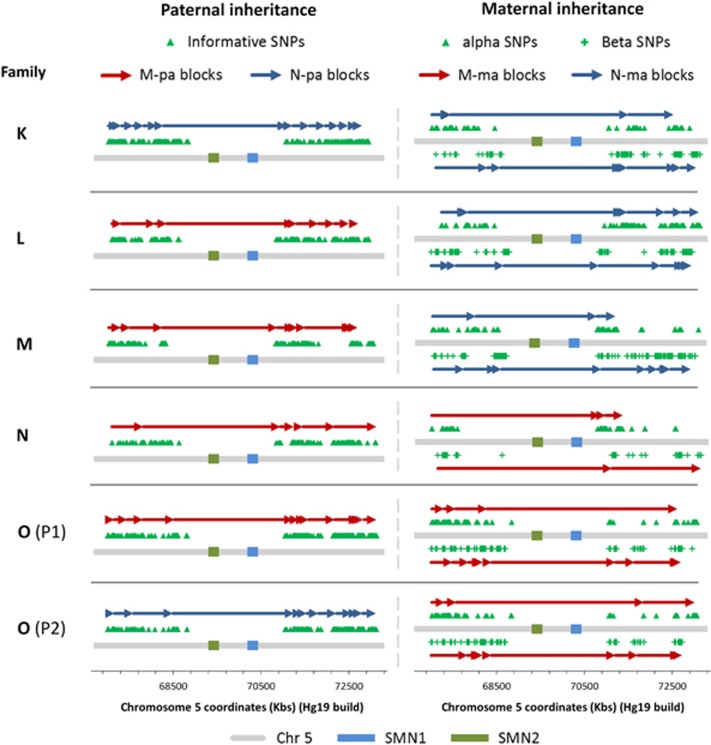
Graphic representation of NIPD results for patients at risk of SMA (families K–O) subdivided by foetal inheritance of paternal and maternal haplotypes. Haplotype blocks are represented by contiguous arrows spanning a 6 Mb genomic window on chromosome 5 (grey line) containing the SMN1 (blue box) and SMN2 (green box) genes. Red arrows indicate that the foetus has inherited the mutated allele (M-pa from the father; and M-ma from the mother), while blue arrows indicate that the normal allele has been inherited (N-pa from the father; and N-ma from the mother). The position of informative SNPs used to identify haplotype blocks is shown for both paternal (green triangles) and maternal (green triangles for *α* SNPs; green crosses for *β* SNPs) inheritance.

Testing of six pregnancies in five patients at risk of carrying a child affected with SMA was conducted for clinical validation. In all cases, the haplotypes linked to the SMA mutations were identified from the DNA sample of a previous affected sibling of the foetus. A summary of results and testing parameters is presented in Table 2, and a graphical representation of maternally and paternally inherited haplotype blocks is illustrated in Figure 2 for each pregnancy. In four of the five patients tested (families K, L, M and N), both parents were confirmed carriers with one copy of exons 7 and 8 in the SMN1 gene detected by MLPA analysis. In family O, only the mother was confirmed as a carrier of SMA (see following section). NIPD of SMA showed an unaffected outcome in family K, carrier status in family L (paternally inherited), carrier status in family M (paternally inherited), affected outcome in family N and an affected and carrier (maternally inherited) results, respectively, for the first (P1) and second (P2) pregnancies in family O. All outcomes were confirmed by MLPA analysis on CVS samples, except for the second pregnancy in family O (P2) (see following section). Testing performances for these patients matched closely with the data obtained from group 1 patients, with an average number of 644 informative SNPs identified (IQR=124) after quality filtering. Foetal fraction ranged from 9.09 to 16.49% with an average of 11.75% and the accuracy of haplotype block classification was 99.26% (135/136), with only one haplotype block incorrectly classified in family K. This data prove the clinical validity of our NIPD test for SMA, which reported a sensitivity and specificity rate of 100% for patients tested with no inconclusive results and a failure rate of 0%.

### Advantages of NIPD over invasive testing in family O

**Figure 3:**
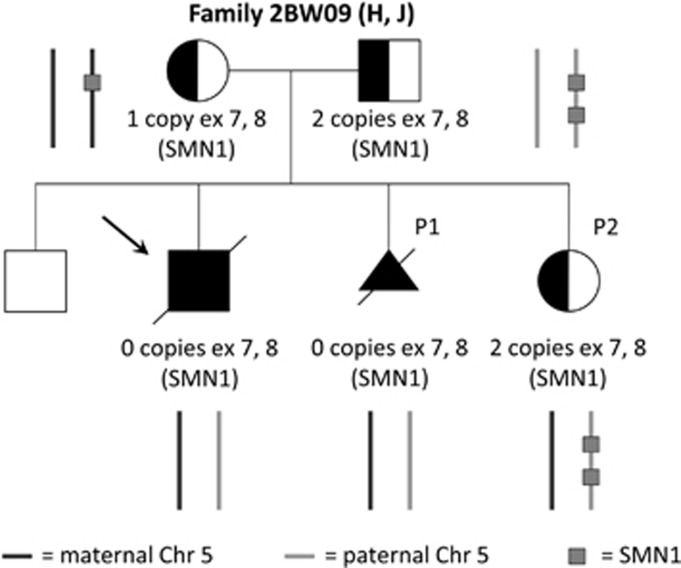
Family tree of family O. Copy number status of the SMN1 gene is reported for family members affected by/carriers of SMA, as reported by MLPA analysis. This is graphically illustrated by showing the copies of SMN1 gene for both maternal (black) and paternal (grey) alleles on the side. A DNA sample from the previous affected son (black arrow) was used to identify the maternal and paternal haplotypes linked with the mutant alleles. NIPD testing for SMA was carried out on the subsequent pregnancies (P1 and P2).

The parents in family O were screened for SMA carrier status after their second child was diagnosed with the disease (Figure 3). The mother was found to be carrying one copy of exons 7 and 8 in the SMN1 gene, as detected by MLPA analysis. The carrier status of the father, however, could not be confirmed, as he was found to have two copies of exons 7 and 8 for SMN1. Therefore, it was hypothesized that the father may be carrying two copies of SMN1 on one allele and none on the other, as has been proven to happen in around 4% of cases.^32^ Alternatively, the father may have been a carrier for a mosaic germline mutation or there may have been a *de novo* mutation on one allele in the affected child. NIPD testing on the patient's subsequent pregnancy (P1) using the DNA sample from the previous affected sibling of the foetus to identify the haplotypes linked with the mutant alleles yielded an affected result (Figure 2). The outcome was confirmed by MLPA analysis conducted on the CVS sample and indicated, therefore, that the father was likely to be a carrier of SMA. In the following pregnancy (P2), NIPD testing revealed that the foetus was a carrier, having inherited the mutated haplotype from the mother (M-ma) and the normal haplotype from the father (N-pa; Figure 2). In this case, MLPA analysis conducted on the invasively obtained foetal sample yielded an unaffected result, having detected two copies of exons 7 and 8 in the SMN1 gene. Taken together, this data prove that the father is indeed a carrier of SMA and has two copies of SMN1 on one allele and none on the other, as previously hypothesized. In summary, NIPD testing of SMA in this family provided a more informative result by confirming the carrier status of the second tested pregnancy (P2). Although the foetus would have still been correctly diagnosed as unaffected by invasive testing, knowledge of its carrier status holds important implications for the baby's future reproductive possibilities. However, it is important to highlight that, in cases where carrier status is not confirmed for one of the parents, a confirmatory invasive test would need to be conducted for affected outcomes in which the high-risk haplotype has been identified by NIPD testing. This is because we are testing for a haplotype and not the mutation directly. Therefore, pregnancy P1 would have been reported as ‘increased risk' rather than ‘affected' in a clinical setting, and an invasive test would have been warranted to confirm the ‘affected' outcome.

## Discussion

The implementation of NIPD tests for SGDs into clinical practice has been slow and limited. By comparison, foetal sexing and RhD typing using cffDNA are now in routine clinical service^11^ in many countries across Europe. Furthermore, non-invasive aneuploidy screening tests developed by several commercial companies^13, 35, 36, 37^ are being introduced in public health services worldwide,^16^ yet only a few centres are offering NIPD for SGDs. In the United Kingdom, a number of NIPD tests for the detection of paternally inherited and *de novo* mutations in selected SGDs have been developed as part of the RAPID project,^21, 22^ some of which are now available as a clinical service.^11^ Although various studies have demonstrated that comprehensive NIPD testing for certain SGDs is technically possible,^23, 24, 25, 26, 27^ challenges pertaining to the high testing costs incurred remain to be addressed before clinical implementation can be achieved.^24, 26^ Recently, our team at Birmingham Women's NHS Foundation Trust has succeeded in developing an affordable and clinically feasible NIPD test for DMD/BMD through the NIPSIGEN project.^28^ Building on our experience, we have endeavoured to develop similar tests for other common autosomal-recessive disorders. In this study, we describe a new method for the NIPD of SMA by RHDO analysis capable of delivering accurate and informative results, as well as the foetus' SMA carrier status, irrespective of the SMA mutation profile present in the parents. This is an advantage in cases where copy number analysis of the SMN1 gene in carrier screening and prenatal tests is unable to confirm carrier status owing to a parental allele carrying two copies of the gene. However, in these cases NIPD tests revealing an affected outcome would need to be confirmed by an invasive test to avoid the possibility of a false positive result owing to germline mosaicism in the parent with unconfirmed carrier status or the possibility of a *de novo* mutation.

Overall, the data obtained from all the 13 patients tested, of which six pregnancies at risk of SMA, has shown sensitivity and specificity rates of 100%, with 0% failure rate. The accuracy of our method in correctly classifying haplotype blocks amounted to 99.43% (351/353). This reflects the reliability of the test, as each haplotype block represents a statistically independent result. Although we did not detect any recombination events across the targeted region containing the SMN1 and SMN2 genes in patients tested so far, the numbers of haplotype blocks identified for both paternal and maternal inheritance led us to believe that our method would be capable of detecting recombination sites with high accuracy. However, this would not apply to recombination events occurring within the highly repetitive genomic region on chromosome 5 located around the SMN1 and SMN2 genes (chromosome coordinates: 68 813 676 and 70 680 481) (Figure 1), where we were unable to design unique probes for targeted enrichment of selected SNPs. Although the possibility of this event is <1%,^38^ an inconclusive result would be reported in these cases and prenatal diagnosis by invasive testing would be warranted. Additionally, our NIPD test is not applicable in dizygotic twin pregnancies and in the absence of a viable DNA sample from an affected sibling. There also remains a very small risk of misdiagnosis in the event of a vanishing twin or in the presence of a double recombination within the highly repetitive region containing the SMN1 and SMN2 genes where we were unable to design SNP probes. Other limitations of our NIPD test include the common limitations associated with cffDNA analysis, such as the impossibility of obtaining a viable diagnostic result in the presence of maternal somatic mosaicism or if the patient has undergone transplant surgery (ie, is the recipient of a donor organ). Low foetal fraction in cfDNA is also a limitation to NIPD testing, with 4% foetal fraction being generally considered as the cutoff limit.^39, 40, 41^ Nevertheless, data published in a previous study^24^ and generated in our laboratory (data not shown) led us to believe that a foetal fraction of 2% would reflect a more accurate cutoff limit for NIPD testing by RHDO analysis. In fact, the quantity of informative SNPs used for data analysis ensures that a statistically significant result is reached even in the presence of low foetal fractions. This, however, might not be the case for pregnancies in consanguineous couples, where the quantity of informative SNPs usable for RHDO analysis would be significantly reduced. In these cases, SNPs that are heterozygous in both parents would become informative and could be used to determine the maternally and paternally inherited haplotypes,^23^ although further testing on consanguineous couples would be required to validate this analysis method for clinical use.

Implementation of NIPD testing for SGDs into clinical practice has been mainly hindered by the prohibitive testing costs incurred,^24, 26^ which cannot be curbed by increasing the patient multiplexing capacity of the test owing to the small number of couples who would request it. In this respect, the method presented in this study is capable of delivering accurate NIPD of SMA at an arguably reasonable cost. This is mainly achieved by designing highly efficient capture probes for targeted enrichment, which enables the use of lower-cost MiSeq sequencing platform (Illumina, Inc.) sequencing platform; by being able to multiplex up to three patients on the same sequencing run for up to three different SGDs; and by testing maternal, paternal and proband samples alongside the cfDNA sample to reduce haplotyping costs. Taking these considerations into account, we have calculated the laboratory cost of the test to be £585 per patient (consumables and staff costs only) when multiplexing three patients. However, the full cost would need to take into account equipment costs and additional overheads. An additional cost that is impossible to quantify is the influence that NIPD testing might have on patient choice. Cost to a health service would potentially be increased by couples requesting non-invasive tests, which they would not have considered pursuing by invasive techniques. This needs to be weighed against the increased number of positive prenatal diagnoses and the financial impact of subsequent management decisions. Furthermore, consideration needs to be given to the quality improvement gained from increased patient choice and avoidance of procedure-related miscarriage. We therefore believe the final cost of the test to be acceptable for clinical use in the United Kingdom and are in the process of seeking approval from the UK Genetic Testing Network (UKGTN) to recommend national commissioning within the NHS. By running the test on a weekly basis with a turn-around time of 7–10 working days (depending on the day of the week the blood sample is received), we aim to provide the patient with a final report within the first trimester of pregnancy, as the blood sample can be taken as early as 8 weeks' gestation. Therefore, our NIPD method will not only deliver a safer test by eliminating the risk of miscarriage linked with invasive sampling procedures, thus improving quality of service, but will also provide patients with more time to manage their pregnancy.

In summary, having previously proved that NIPD of an X-linked condition can be achieved by RHDO analysis in a clinical setting,^28^ we here provide evidence that the same approach can be applied to autosomal-recessive disorders. Initial validation of our NIPD test for SMA has shown promising results, with an overall sensitivity and specificity of 100% on 16 patients tested to date. Given our success in using RHDO analysis for NIPD of SGDs, we intend to implement such testing into clinical practice within the United Kingdom and widen our testing repertoire, with the aim of enabling our patients to access swift and safe NIPD testing for a variety of Mendelian disorders.

## Figures and Tables

**Table 1:** Summary of tests conducted on patients from group 1 (pregnancies A–J)

*Pregnancies*	*Outcome (mat/pat)*	*Gestation*	*Foetal fraction*	*Reference haplotypes*	*Informative SNPs used*	*Haplotype blocks (mat/pat)*	*Classification accuracy*	*Average sequencing depth of informative SNPs used*
A	HapA–HapA	13 weeks+4 days	14.34%	CVS	823	18/8	100%	339
B	HapA–HapA	13 weeks+5 days	7.56%	CVS	898	20/12	100%	321
C	HapA–HapA	14 weeks+5 days	9.83%	CVS	726	18/10	100%	259
D	HapA–HapA	13 weeks+1 day	7.51%	CVS	811	13/11	100%	270
E	HapA–HapA	11 weeks+2 days	20.31%	CVS	566	14/8	100%	223
F	HapA–HapA	15 weeks+5 days	6.83%	AMNIO	675	10/10	95%	257
G	HapA–HapA	13 weeks+5 days	11.69%	CVS	653	14/10	100%	189
H	HapA–HapA	11 weeks+1 day	12.21%	CVS	531	11/9	100%	149
I	HapA–HapA	11 weeks+3 days	6.91%	CVS	278	3/5	100%	89
J	HapA–HapA	14 weeks	12.10%	CVS	360	9/4	100%	64

Outcomes are reported as paternal and maternal inheritance of the reference haplotypes (HapA) identified in the foetal DNA obtained by invasive sampling or the opposite haplotypes (HapB) identified in the maternal and paternal DNA samples by haplotype phasing. Only informative SNPs that passed the quality filtering criteria (see Supplementary Appendix A) were used for data analysis. The numbers of haplotype blocks identified for paternal and maternal inheritance are kept separate. The classification accuracy represents the percentage of haplotype blocks, which showed an expected inheritance pattern. The average sequencing depth has been calculated on the informative SNPs used for data analysis.

**Table 2:** Summary of tests conducted on patients from group 2 (families K–O)

*Family*	*Maternal mutation in SMN1*	*Paternal mutation in SMN1*	*NIPD outcomes (mat/pat)*	*Gestation*	*Foetal fraction*	*Invasive PND outcomes*	*Informative SNPs used*	*Haplotype blocks (mat/pat)*	*Classification accuracy*	*Average sequencing depth of informative SNPs used*
K	1 copy of exon 7	1 copy of exon 7	Unaffected (N-ma/N-pa)	11 weeks+3 days	9.09%	Unaffected	803	12/14	96%	274
L	1 copy of exons 7 and 8	1 copy of exon 7 and 8	Carrier (N-ma/M-pa)	12 weeks	10.67%	Carrier	685	18/9	100%	161
M	1 copy of exons 7 and 8	1 copy of exons 7 and 8	Carrier (N-ma/M-pa)	11 weeks+5 days	10.91%	Carrier	592	12/10	100%	174
N	1 copy of exons 7 and 8	1 copy of exons 7 and 8	Affected (M-ma/M-pa)	12 weeks+4 days	9.98%	Affected	370	5/6	100%	86
O (P1)	1 copy of exons 7 and 8	2 copies of exons 7 and 8	Affected (M-ma/M-pa)	11 weeks+2 days	13.35%	Affected	751	13/13	100%	252
O (P2)	1 copy of exons 7 and 8	2 copies of exons 7 and 8	Carrier (M-ma/N-pa)	13 weeks+1 day	16.49%	Unaffected	665	12/12	100%	158

Maternal and paternal SMA mutations for both parents were known from carrier screening tests conducted by MLPA analysis. Outcomes are reported as maternal and paternal inheritance of the haplotypes linked to the mutated (M-ma, M-pa) or normal (N-ma, N-pa) alleles. Prenatal diagnosis (PND) on the invasively obtained sample was conducted by MLPA analysis.
