# Supplementary material for: Non-invasive prenatal diagnosis of spinal muscular atrophy by relative haplotype dosage
Source: Eur J Hum Genet. 2017 Jan 25;25(4):416–22. doi: 10.1038/ejhg.2016.195 (PMC5386415; doi:10.1038/ejhg.2016.195)
Supplement: Supplementary Materials [file ejhg2016195x1.docx]

**SUPPLEMENTARY**

**Appendix A: sample collection and processing**

Patient recruitment was conducted through the NIPSIGEN study (“NIPSIGEN: clinical translation of NIPD for single gene disorders”; REC approval number: 13/NW/0580). Pregnant women referred to West Midlands Regional Genetics Laboratory for increased risk of aneuploidies were recruited to group 1; pregnant women and their partners who carried an SMA mutation were recruited at a national level (UK) to group 2. All patients were requested to donate 20 ml of peripheral blood prior to CVS (see table 1 for details of gestational age at blood draw) which was collected in EDTA tubes for group 1 patients and Cell-Free DNA BCT tubes (Streck) for group 2 patients. Plasma isolation was conducted within 24 hours of bleeding for EDTA tubes and 72 hours for Cell-Free DNA BCT tubes. Blood samples were centrifuged at 1600 g for 10 minutes; isolated plasma was further centrifuged at 16,000 g and stored at -80˚C. The blood cell portion was stored at -20˚C. CfDNA was extracted from 4 ml of plasma on the automated Qiasymphony platform (Qiagen) using the QIAsymphony DSP Virus/Pathogen Midi kit and following the custom modified protocol for DNA extraction from large volumes of sample. DNA was eluted in a final volume of 60 µl. Maternal and paternal genomic DNA was extracted from the leukocytes contained in 1 ml of the blood cell portion (post plasma isolation) using the QIAamp Blood Mini kit on the automated Qiacube platform (Qiagen) following the supplier’s protocol. Genomic DNA from CVS and amniotic fluid samples was extracted with the EZ1 DNA Tissue kit on the automated EZ1 platform (Qiagen). Extracted DNA concentration was measured using the Qubit 2.0 High Sensitivity kit (Invitrogen).

**Appendix B: targeted MPS**

Genomic DNA from maternal, paternal, proband, CVS and amniotic fluid samples was sheared to a length of 180-220 bp on the Covaris M220. CfDNA does not require shearing as it is already fragmented in nature[1]. 29-122 ng of input DNA per sample was used to prepare libraries for sequencing on the Illumina MiSeq using the KAPA Hyper Library Preparation kit for Illumina platforms (KAPA biosciences). Targeted capture enrichment was performed using a custom SeqCap EZ Choice probe library (Nimblegen) to enrich for DNA fragments containing SNPs with average heterozygosity > 0.4 across the SMN1 and SMN2 genes region on chromosome 5 (SMN1: 70,220,768-70,248,839; SMN2: 69,345,350-69,373,422; hg19 build). The total captured area was 276 Kb at 67% probe coverage. 8-12 samples (equivalent to 2-3 patients) were multiplexed per sequencing run on the MiSeq (Illumina) using the 150 cycle PE V3 kit. The settings used included single indexing and 2x80 cycles paired end sequencing.

**Appendix C: bioinformatics pipeline**

Trimmomatic v0.32[2] was used to quality trim reads that fell below a quality score of 30.  Bowtie2 v2.1.0[3] was used to align trimmed reads to the human reference genome, hg19, and a combination of Picard tools v1.97[4], SAMtools v0.1.19[5] and GenomeAnalysisTK v2.7-4-g6f46d11[6–8] were used for pre-processing and duplicate removal. Variant calling of known SNPs in the region was carried out by platypus v.0.6.0[9].

**Appendix D: RHDO analysis parameters and fetal fraction calculation**

RHDO analysis was applied in the current study as described in previous publications[10, 11]. Phasing of maternal, paternal and proband (or CVS/amnio) haplotypes was conducted using Excel worksheets (Microsoft Office 2010). Informative SNPs were required to show a sequencing depth ≥ 30 (in genomic DNA and cfDNA samples) and be separated from each other by ≥ 200 bp, in order to reduce bias[11, 12]. Haplotype blocks had to contain ≥ 25 informative SNPs, to minimize stochastic influences. For a recombination event to be called, two or more consecutive haplotype blocks showing a switch in fetal inheritance had to be observed[10]. An odds ratio of 1200 was used in SPRT classifications[10] for maternally inherited haplotype blocks. RHDO analysis on α and β SNPs was conducted separately, as outlined in previous publications[11, 13]. Statistical significance of paternally inherited haplotype blocks was calculated using the Two Sample Kolmogorov-Smirnov (KS) test, with a cut-off p-value of 0.001[11]. SPRT and KS test calculations and graphical representations of RHDO and KS test analysis outcomes were obtained using Excel worksheets. Fetal fraction was calculated using SNPs that were identified to be homozygous in both parents, but for different alleles, as outlined in previous publications[11, 13]:

cffDNA % = ∑ 2p / ∑ (p+q)

p = read count of the fetal-specific paternal allele

q = read count of the maternal and fetal-specific maternal allele

**REFERENCES**

1 Chan KCA. Size Distributions of Maternal and Fetal DNA in Maternal Plasma. *Clin Chem* 2004;**50**:88–92.

2 Bolger AM, Lohse M, Usadel B. Trimmomatic: a flexible trimmer for Illumina sequence data. *Bioinforma Oxf Engl* 2014;**30**:2114–20.

3 Langmead B, Salzberg SL. Fast gapped-read alignment with Bowtie 2. *Nat Methods* 2012;**9**:357–9.

4 *Picard.* http://broadinstitute.github.io/picard/ (accessed 28 Aug2015).

5 Li H, Handsaker B, Wysoker A, Fennell T, Ruan J, Homer N, Marth G, Abecasis G, Durbin R, 1000 Genome Project Data Processing Subgroup. The Sequence Alignment/Map format and SAMtools. *Bioinforma Oxf Engl* 2009;**25**:2078–9.

6 McKenna A, Hanna M, Banks E, Sivachenko A, Cibulskis K, Kernytsky A, Garimella K, Altshuler D, Gabriel S, Daly M, DePristo MA. The Genome Analysis Toolkit: a MapReduce framework for analyzing next-generation DNA sequencing data. *Genome Res* 2010;**20**:1297–303.

7 DePristo MA, Banks E, Poplin R, Garimella KV, Maguire JR, Hartl C, Philippakis AA, del Angel G, Rivas MA, Hanna M, McKenna A, Fennell TJ, Kernytsky AM, Sivachenko AY, Cibulskis K, Gabriel SB, Altshuler D, Daly MJ. A framework for variation discovery and genotyping using next-generation DNA sequencing data. *Nat Genet* 2011;**43**:491–8.

8 Van der Auwera GA, Carneiro MO, Hartl C, Poplin R, Del Angel G, Levy-Moonshine A, Jordan T, Shakir K, Roazen D, Thibault J, Banks E, Garimella KV, Altshuler D, Gabriel S, DePristo MA. From FastQ data to high confidence variant calls: the Genome Analysis Toolkit best practices pipeline. *Curr Protoc Bioinforma Ed Board Andreas Baxevanis Al* 2013;**43**:11.10.1–33.

9 Rimmer A, Phan H, Mathieson I, Iqbal Z, Twigg SRF, WGS500 Consortium, Wilkie AOM, McVean G, Lunter G. Integrating mapping-, assembly- and haplotype-based approaches for calling variants in clinical sequencing applications. *Nat Genet* 2014;**46**:912–8.

10 Lo YMD, Chan KCA, Sun H, Chen EZ, Jiang P, Lun FMF, Zheng YW, Leung TY, Lau TK, Cantor CR, Chiu RWK. Maternal Plasma DNA Sequencing Reveals the Genome-Wide Genetic and Mutational Profile of the Fetus. *Sci Transl Med* 2010;**2**:61ra91–61ra91.

11 New MI, Tong YK, Yuen T, Jiang P, Pina C, Chan KCA, Khattab A, Liao GJW, Yau M, Kim S-M, Chiu RWK, Sun L, Zaidi M, Lo YM. Noninvasive Prenatal Diagnosis of Congenital Adrenal Hyperplasia Using Cell-Free Fetal DNA in Maternal Plasma. *J Clin Endocrinol Metab* 2014;**99**:E1022–30.

12 Parks M, Court S, Cleary S, Clokie S, Hewitt J, Williams D, Cole T, MacDonald F, Griffiths M, Allen S. Non-invasive prenatal diagnosis of Duchenne and Becker muscular dystrophies by relative haplotype dosage. *Prenat Diagn* 2016;**36**:312–20.

13 Lam K-WG, Jiang P, Liao GJW, Chan KCA, Leung TY, Chiu RWK, Lo YMD. Noninvasive Prenatal Diagnosis of Monogenic Diseases by Targeted Massively Parallel Sequencing of Maternal Plasma: Application to -Thalassemia. *Clin Chem* 2012;**58**:1467–75.
